# Prof. Peebles’ Address

**Published:** 1867-10

**Authors:** 


### Editorial.

### PROF. PEEBLES' ADDRESS.

In the September number of the Register is a long address delivered before the "Chicago Dental Society." This was unseen by one of the editors (till in print), and unread by the other, or it would have been accompanied with a few remarks. Contrary to rule, it was inserted on the credit of its respected author and the society which officiated at its delivery. If it should now be referred to at a greater length than would have been thought necessary at the time of its appearance, its author and friends will not complain. We do not propose to devote a score of pages to its consideration. We have given that much to its insertion.

The address is on "Education," and bears about the same relation to the "Missouri Dental College" that McLean's Almanac does to the "Strengthening Cordial." Much of the address is good; and but little of it is positively objectionable. The same is true of the almanac: its calculations of eclipses are reliable; it tells the rising and setting of the sun; it gives the changes of the moon; its picture of the man with a ram above him, and a lion beside him is good; and some of its "original anecdotes" have not been printed more than fifty times.

The character of the address is consistent with its authorship. Had it appeared as a late production of the sainted Harris, it would have taken the profession by surprise. He had no such positive notions on the subject of professional education. Indeed, he was so befogged by the mists of special science as to promulgate the "Principles and Practice of *Dental* Surgery." Others, too, who like him, have labored for years as educators of our profession, are overwhelmed with doubts, and beset by misgivings as to the true theory of professional instruction. But a new professor, just elected, has none of these doubts. If called on by any society before he has been spoiled by delivering a course of lectures, he can tell just how the thing is to be done. At that stage of his professorial career he knows all that is worth knowing about the education of the profession. And this position is not to be disputed, not even doubted; for we speak from experience. Why, a dozen or more years ago we could have told the old "American Society" all it wanted to know, and would have done so, if it had asked us! Now we are despondent with dread that our next course of lectures will prove a total failure.

This address sets out with the proposition that "A specialty can not be separated from the parent stock, and be maintained as an independent profession," which is simply "begging the question," or assuming as true the matter in dispute. Every one will admit that a specialty in medicine can not be maintained independently of *medical science;* but that it can not be maintained independently of the *medical* profession, as such, is another matter. A specialty in agriculture can not ignore soil and climate; but sugar-making and cotton-raising may be carried on independently of each other. The soil and the climate belong to the one as well as to the other. And the boy destined to become a cotton planter is not the best educated by confining him, through early life, to raising cane, tobacco, corn, hemp, wheat, etc., only allowing him occasionally to see a cotton seed or a cotton plant, so that when he takes possession of his own estate he may know what to plant. True, this would be better than no education, for he would thus learn to plow, and would develop his muscles, but he would accomplish these ends quite as well in raising cotton, and would, at the same time, gain a knowledge of the characteristics of the plant which is to be the basis of his fortune.

This figure is not overdrawn, and applies well to the case in hand. The author of the address is a professor in the "Missouri Dental College," at St. Louis, which he modestly tells us is "not least" among the colleges.

("If self the wavering balance shake
It's rarely right adjusted")

Now, for lack of material, want of harmony, diversity of sentiment as to the necessity of another college, or some other reason, the Dental profession was not in condition to carry on a college in that locality, without aid from the medical profession. It was their duty to obtain this aid. And if their medical co-operators would prepare their lectures with direct reference to the wants of Dental Surgery (for there is such a thing), all would be well. But the physicians in the Missouri Dental (?) College lecture to train *physicians*, not Dentists. The blacksmith shop is not the best place to learn the carpenter trade, even though there may be a good deal of hammering done in it.

The mother of eleven little porklings had but ten mammary appendages for their accommodation. On the principle that might gives right, a teat apiece was taken by the stoutest, leaving a slim little fellow unprovided for. He seized the tail for want of something better, and sucked and starved, and starved and sucked; but in his faint dying squeals he didn't advise any of his comrades to play the part of *the eleventh pig*. Yet this is the part played by the Dental student when listening to lectures adapted to the training of general practitioners. And when they have taken the regular course in a medical college they know as little of Dentistry as the young planter above referred to does of cotton-raising. He knows the cotton plant when he sees it; they are not likely to know a lower from an upper molar, after extraction. In our own medical course, all that was taught us, in the lecture room, in reference to the teeth, might have been taught in two hours, if not in one. Such was all that anatomy, physiology, pathology, chemistry, therapeutics and surgery could afford us on these organs, while so many others demanded attention.

We have already stated that much of the address is good. The Dental profession will not believe that we favor a narrower range of information than that advocated by it. And had it avoided the slanders implied or expressed in its special pleadings, we would have been saved the trouble of writing this.

The author asks, "Can any specialty in medicine be fully acquired, or thoroughly taught in a school, when the general principles of medical science are not taught in the fullest sense of the term?"

To this we would reply, the experiment has never been tried, only that would bring us in direct issue with the author; for he goes on to say, " The science of medicine, in its general range, is not taught in our schools of specialty, as they are generally constituted. Nor will it be; so long as the absurdity of making a *mere* specialist is kept up, and our schools ignore the fact that a thorough knowledge of general anatomy, physiology, pathology and therapeutics is essential to the education of our specialists."

" *Our* schools of specialty" are the Dental colleges. The statement is that the science of medicine in its general range is not taught in these; and that they ignore the fact that general anatomy, physiology, etc., are essential parts of Dental education.

These are taught in the Missouri College it is claimed; and herein is the *essence* and *spirit* of the address. This is the calling " special attention to strengthening cordial;" and we have no objections to the cordial, but when it is charged that the " genuine old Dr. Jacob" doesn't keep good sarsaparilla, we must be allowed to respond.

If the author of the address would take another course with his *alma mater*, or one of her *sisters*, he would retract this charge, and modify a number of his statements.

Schools of general medicine had a fair trial before Dental schools were organized. So far as teaching Dental surgery is concerned they *utterly failed*. They would fail again, and for the same reasons. Dental science was the *eleventh pig*. Its votaries had no chance. Occasionally an energetic student forced a little special instruction from the general mass, just as the extra pig, when stout enough, occasionally drops the tail and snatches a teat, when its rivals are not watching. Such a student was Dr. Harris, who finding, that for sucking purposes, teats are vastly better than tails, determined that his younger brethren should be furnished with " sincere milk" of Dental science, and so inaugurated the era of DENTAL COLLEGES. These have done more, in a score of years, to advance Dental science and practice, than the medical schools have done since the days of Hippocrates. That they have done well is admitted even by the author of the address. He says, " We are far advanced in the art, but can the art precede the science?" He also quotes some one to prove that art can not go ahead of science. He says too, that we have made as rapid progress in knowledge and science as the medical profession. He claims for our specialty an "unprecedented advancement;" and he mentions our Dental colleges as prominent agencies in producing this advancement; but now he proposes to go back to the old way, relying mainly on physicians to teach Dentistry.

A fox lost his tail in a trap, and advised his comrades to cut their tails off; but they didn't do it. A little friend of ours had weak ankles. They were kept straight by steel stays. He became almost too proud to play with children who didn't need bracing. We are glad the Missouri College has started. We hope its friends will make it a *Dental* college as soon as practicable—that they will take off its braces as soon as it can walk alone. W.

